# NoTox Tweakments: Evidence for Topical Neurocosmetic Peptides as Needle‐Free Neuromodulators for Facial Wrinkle Reduction—A Systematic Review

**DOI:** 10.1111/jocd.71137

**Published:** 2026-08-25

**Authors:** Phyo Zaw Aung, Thamthiwat Nararatwanchai

**Affiliations:** ^1^ School of Anti‐Aging and Regenerative Medicine Mae Fah Luang University Bangkok Thailand

**Keywords:** botulinum toxin‐like peptides, neurocosmetics, NoTox Tweakments, wrinkle reduction

To the Editor,

“NoTox Tweakments” is a term that captures an emerging class of topical neurocosmetic peptides designed to mimic the wrinkle‐relaxing mechanism of botulinum toxin type A (BoNT‐A) without the need for injections. The name derives from the combination of *nontoxic*, *non‐injection*, and tweak‐level aesthetic adjustment. As patient demand for needle‐free aesthetic procedures continues to grow, compounds such as acetyl hexapeptide‐8 (AHP‐8, Argireline), Leuphasyl, Syn‐Ake, and Myoxinol have entered the cosmeceutical market with claims of SNARE‐complex inhibition and consequent reduction of dynamic and static facial wrinkles. Despite commercial proliferation, a rigorous synthesis of their evidence base relative to the established gold standard of injectable BoNT‐A has been lacking.

Injected BoNT‐A represents the current gold standard for dynamic facial wrinkle reduction. In pivotal phase III trials, a single treatment with onabotulinumtoxinA (20 U) achieved responder rates of 63.9%–89.5% at day 30 for glabellar lines and crow's feet, with effects sustained for approximately 3–6 months before reinjection is required [1, 2]. Its mechanism involves complete, localized neuromuscular blockade, producing rapid paralysis of targeted mimetic muscles. However, injectable BoNT‐A carries known limitations: requirement for trained administration, risk of ptosis or adjacent muscle paralysis, contraindications in pregnancy, and the need for repeated procedures every 3–5 months to maintain effect.

We conducted a PRISMA 2020‐compliant systematic review registered with PROSPERO (CRD420261405299) to evaluate the efficacy and safety of topical botulinum toxin‐like peptides. We searched PubMed, Scopus, Web of Science, and CENTRAL from inception to June 2026, retrieving 847 records. After deduplication and screening, 11 studies met eligibility criteria: 7 randomized controlled trials, 3 prospective observational studies, and 1 single‐group uncontrolled trial, enrolling 412 participants total. The PRISMA flow is presented in Figure 1.

**FIGURE 1:**
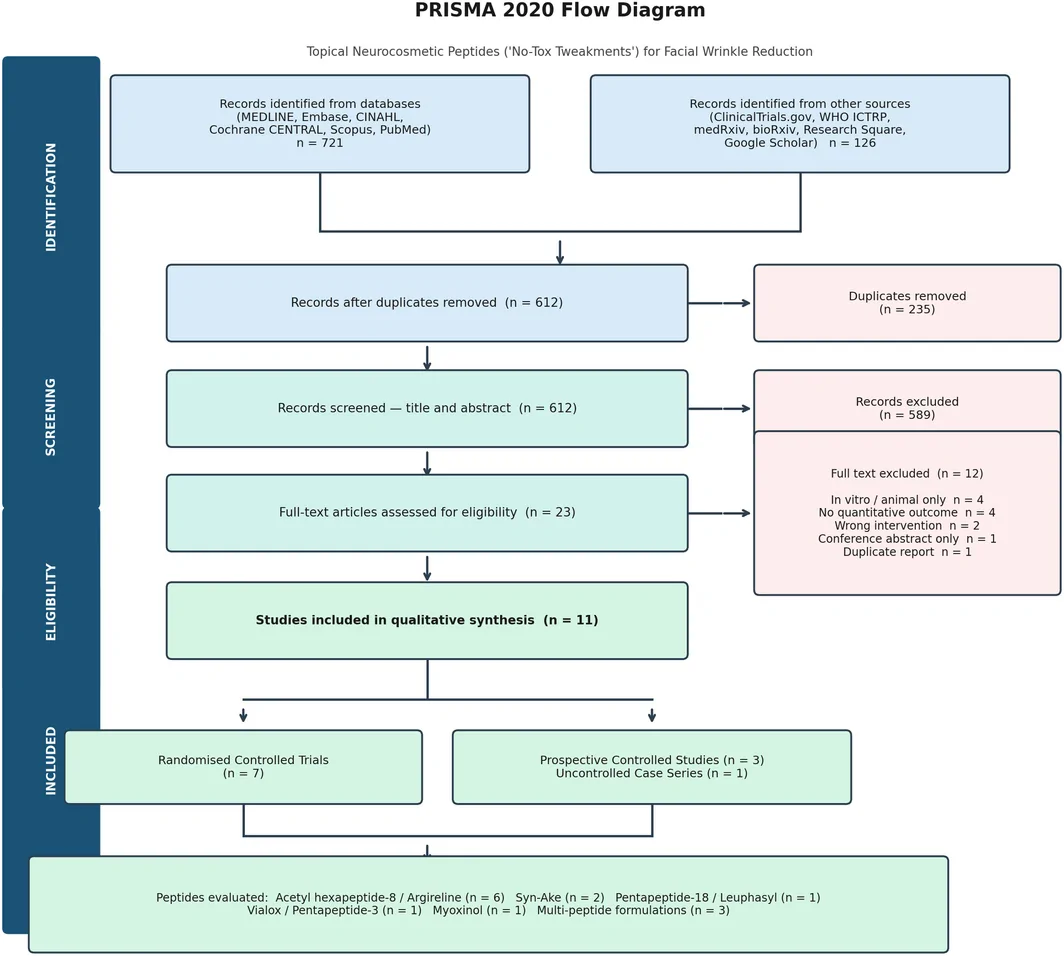
The PRISMA 2020 flow diagram.

Efficacy findings were clinically meaningful, though quantitatively more modest than injectable BoNT‐A. AHP‐8 at 10% concentration produced a 48.9% reduction in periorbital wrinkle depth versus 0% in placebo controls at 4 weeks (*n* = 60) [3]. A multi‐peptide serum demonstrated a 100% responder rate for fine‐line improvement and 67% for deeper wrinkles versus placebo at 12 weeks (*p* < 0.001; *n* = 56) [4]. A dual‐peptide serum combining AHP‐8 and Syn‐Ake achieved a 35%–69% improvement in static wrinkles and 10%–13% reduction in dynamic wrinkle depth at 12 weeks [5]. These gains are lower in absolute magnitude than the 64%–90% responder rates achieved with injectable BoNT‐A at 4 weeks [1, 2], yet are particularly notable for static wrinkles, which injectable BoNT‐A addresses less effectively [6]. Topical peptides also offer continuous, cumulative benefit through daily use, contrasting with the discrete on/off profile of injectable treatment. Key results are summarized in Table 1.

**TABLE 1:** Comparison of injectable Botulinum toxin A (standard) with topical neurocosmetic peptides (NoTox Tweakments) for facial wrinkle reduction.

Treatment	*n*	Duration	Primary outcome	Effect vs. placebo	Safety	Study type	RoB
Injectable BoNT‐A (onabotulinumtoxinA 20–24 U) [standard] [1]	317–417	4 weeks	Crow's feet/glabellar lines at max frown	64%–90% responder rate	Ptosis 5.4%; reinjection q3‐5 months	Phase III RCTs [1, 2]	Low
AHP‐8 10% cream (topical) [3]	60	4 weeks	Periorbital wrinkle depth	48.9% vs. 0% placebo	No AEs	RCT	Moderate
Multi‐peptide serum (AHP‐8 + HA) [4]	56	12 weeks	Fine‐line and wrinkle improvement	100%/67% responders (*p* < 0.001)	No AEs	RCT, DB	Low–moderate
AHP‐8 + Syn‐Ake serum (topical) [5]	50 + 42	12 weeks	Static and dynamic wrinkles	35%–69% static; 10%–13% dynamic (*p* < 0.001)	No AEs	Prospective	Moderate–high
AHP‐8 microneedle patch (transdermal patch)^a^ [7]	52	4 weeks	Periorbital wrinkle + hydration	Patch superior to patch alone (*p* < 0.05)	No AEs	RCT, split‐face	Moderate
BMa/AHP‐8 ionic liquid cream (topical) [6]	N/R	28 days	Wrinkle number, length, area	3.1× improved penetration; greater wrinkle reduction vs. AHP‐8 alone	No AEs	RCT	Moderate
Novel nAChR peptide (Medipep, topical) [8]	N/R	6 weeks	Periorbital wrinkles	Significant improvement at 3 and 6 weeks	No AEs	Clinical study	High
Argireline in HA serum (topical) [9]	N/R	Short‐term	Wrinkle score (Visia)	No significant reduction (*p* = 0.829)	No AEs	Controlled split‐face	Moderate–high
Acetyl hexapeptide‐3 (topical) [10]	40	4 weeks	Skin mechanical properties	Significant improvement at 2 and 4 weeks	No AEs	Controlled study	High
Argireline analogues (topical, ex vivo/pilot) [11]	N/A	4 weeks	Wrinkle reduction, skin permeation	Up to 48% wrinkle reduction; improved skin permeation	No AEs	In vitro + pilot	High
Syn‐Ake 4% cream (topical) [manufacturer data]	100	28 days	Wrinkle depth/smoothing	Up to 52% wrinkle reduction; 73% responders	No AEs	Controlled	High
Pentapeptide‐3/Vialox (topical) [manufacturer data]	N/R	28 days	Wrinkle size, muscle contraction	49% wrinkle reduction; 71% reduction in muscle contraction at 1 min	No AEs	In vitro + clinical	High

Abbreviations: AEs, adverse events; AHP‐8, acetyl hexapeptide‐8; BoNT‐A, botulinum toxin type A; DB, double‐blind; HA, hyaluronic acid; N/A, not applicable; N/R, not reported; nAChR, nicotinic acetylcholine receptor; RCT, randomized controlled trial; RoB, risk of bias.

^a^ An et al. [7] used a microneedle patch delivery system; active ingredient is AHP‐8. Results interpreted with this distinction in mind (see text).

Safety profiles were favorable across all 11 included studies. No systemic adverse events were reported. Local reactions, transient erythema, and mild pruritus, occurred in a minority of participants and resolved without intervention. This profile compares favorably with injectable BoNT‐A, which carries a 5.4% incidence of blepharoptosis and rare but documented risks of distant toxin spread at therapeutic doses [1]. Novel ionic‐liquid encapsulation strategies are actively being developed to improve transdermal AHP‐8 delivery, potentially narrowing the efficacy gap with injectable treatment [6].

A limitation of the included evidence warrants acknowledgment. One included study (An et al. [7]) employed AHP‐8 delivered via a cross‐linked hyaluronic acid microneedle patch, a transdermal delivery system that bypasses the stratum corneum and is therefore not strictly topical. This study was retained in the synthesis because the active ingredient (AHP‐8) and the target outcome (wrinkle reduction) are consistent with the neurocosmetic peptide framework; however, its results should be interpreted with this methodological distinction in mind.

In conclusion, this systematic review provides the first consolidated evidence synthesis for “NoTox Tweakments.” Topical neurocosmetic peptides, particularly AHP‐8 and Syn‐Ake, demonstrate statistically significant, clinically relevant wrinkle reduction with an excellent safety record, positioning them as credible needle‐free neuromodulators. While their efficacy does not yet match the rapid, high‐magnitude response of injectable BoNT‐A, their favorable tolerability, accessibility, and specific benefit on static wrinkles make them a scientifically grounded complement, and in appropriately selected patients, a first‐line option for those who decline injection‐based treatment. Standardized, adequately powered head‐to‐head trials with validated outcomes are warranted to further define their positioning against the injectable standard of care.

## Author Contributions

**Phyo Zaw Aung:** conceptualization, PROSPERO registration, systematic literature search, study selection, data extraction, risk of bias assessment, manuscript drafting and revision, corresponding author. **Thamthiwat Nararatwanchai:** independent data extraction, risk of bias assessment, critical review and revision of manuscript.

## Funding

The authors have nothing to report.

## Ethics Statement

This systematic review was conducted in accordance with the PRISMA 2020 guidelines. No ethical approval was required as the study involved secondary analysis of previously published data with no direct human participant recruitment. No patient data, biological samples, or identifiable information were collected or used. The review was prospectively registered with PROSPERO (Registration No. CRD420261405299).

## Conflicts of Interest

The authors declare no conflicts of interest.

## Data Availability

All data supporting the findings of this systematic review are contained in the published studies cited in the reference list. No new primary data were generated. The PROSPERO registration record is publicly accessible at https://www.crd.york.ac.uk/prospero/ (Registration No. CRD420261405299).
